# Strategies for achieving global collective action on antimicrobial resistance

**DOI:** 10.2471/BLT.15.153171

**Published:** 2015-10-13

**Authors:** Steven J Hoffman, Grazia M Caleo, Nils Daulaire, Stefan Elbe, Precious Matsoso, Elias Mossialos, Zain Rizvi, John-Arne Røttingen

**Affiliations:** a Global Strategy Lab, Faculty of Law, University of Ottawa, Fauteux Hall, 57 Louis Pasteur Street, Ottawa, Ontario, K1N 6N5, Canada.; b Manson Unit, Médecins Sans Frontières, London, England.; c International Division, Norwegian Institute of Public Health, Oslo, Norway.; d Centre for Global Health Policy, University of Sussex, Brighton, England.; e National Department of Health, Pretoria, South Africa.; f LSE Health, London School of Economics & Political Science, London, England.; g Yale Law School, Yale University, New Haven, United States of America.; h Environmental Health and Infectious Disease Control Division, Norwegian Institute of Public Health, Oslo, Norway.

## Abstract

Global governance and market failures mean that it is not possible to ensure access to antimicrobial medicines of sustainable effectiveness. Many people work to overcome these failures, but their institutions and initiatives are insufficiently coordinated, led and financed. Options for promoting global collective action on antimicrobial access and effectiveness include building institutions, crafting incentives and mobilizing interests. No single option is sufficient to tackle all the challenges associated with antimicrobial resistance. Promising institutional options include monitored milestones and an inter-agency task force. A global pooled fund could be used to craft incentives and a special representative nominated as an interest mobilizer. There are three policy components to the problem of antimicrobials – ensuring access, conservation and innovation. To address all three components, the right mix of options needs to be matched with an effective forum and may need to be supported by an international legal framework.

## Résumé

Les dysfonctionnements de la gouvernance et du marché à l'échelle mondiale se traduisent par une impossibilité de garantir l'accès à des médicaments antimicrobiens durablement efficaces. De nombreuses personnes s'emploient à pallier ces dysfonctionnements, mais leurs institutions et leurs initiatives manquent de coordination, de direction et de moyens financiers. La création d'institutions, l'élaboration de mesures d'incitation et la mobilisation des parties intéressées font partie des options possibles pour promouvoir une action collective mondiale sur l'accès aux antimicrobiens et sur leur efficacité. Aucune option isolée ne suffira à venir à bout de tous les problèmes associés à la résistance aux antimicrobiens. Parmi les options institutionnelles prometteuses, il convient de mentionner le suivi des étapes importantes et une équipe spéciale interorganisations. Des fonds mis en commun à l'échelle mondiale pourraient être utilisés pour élaborer des mesures d'incitation et un représentant spécial pourrait être chargé de mobiliser les parties intéressées. Le problème des antimicrobiens comporte trois aspects stratégiques: garantir l'accès, la conservation et l'innovation. Pour agir à l'égard de ces trois aspects, il est nécessaire d'associer les options, harmonieusement combinées, à une structure efficace et, peut-être, de les inscrire dans un cadre juridique international.

## Resumen

Los fracasos de gobernanza mundial y de los mercados significan que no es posible garantizar el acceso a medicamentos antimicrobianos de efectividad sostenible. Muchas personas trabajan para solucionar estos problemas, pero sus instituciones e iniciativas no están lo suficientemente coordinadas, guiadas y financiadas. Las opciones para promocionar una acción colectiva global en cuanto al acceso a los antimicrobianos y la efectividad incluyen la creación de instituciones, la elaboración de incentivos y la movilización de intereses. Ninguna opción por sí sola es suficiente para afrontar todos los desafíos asociados con la resistencia a los antimicrobianos. Las opciones institucionales prometedoras incluyen hitos supervisados y un grupo de acción interinstitucional. Se podría utilizar un fondo combinado global para elaborar incentivos y nominar un representante especial como un movilizador de intereses. El problema de los antimicrobianos tiene tres componentes de las políticas: asegurar el acceso, la conservación y la innovación. Para abordar los tres componentes se necesita que la mezcla correcta de opciones se una a un foro efectivo, y podría necesitar el apoyo de un marco legal internacional.

## ملخص

يؤدي فرض الرقابة العالمية ووقوع أوجه قصور في الأسواق إلى عدم إمكانية ضمان تيسير سبل الحصول على الأدوية المضادة للميكروبات ذات الفعالية المستدامة. وهناك جهود تبذلها جهات عدّة للتغلب على هذه الأوجه من القصور، إلا أن المؤسسات والمبادرات التي تمثلها تلك الجهات تعاني من عدم كفاية التنسيق وقصور القيادة ونقص التمويل. ومن بين الخيارات المتوفرة لتشجيع التحرك الجماعي على المستوى الدولي لتوفير سبل الحصول على الأدوية المضادة للميكروبات وضمان فعاليتها يأتي بناء المؤسسات، وتقديم الحوافز، وغرس الاهتمام. ليس ثمة خيار أوحد يكفي بمفرده لمواجهة جميع التحديات المرتبطة بمقاومة الميكروبات. أما الخيارات المشجعة على مستوى تحرك المؤسسات، فتشمل تخطيط مراحل تخضع للمتابعة وتشكيل قوة عمل تجمع بين الجهات المختلفة. كما أنه من الممكن الاستعانة بصندوق يستمد مصادره من جهات عالمية لتقديم الحوافز، ويمكن أيضًا ترشيح ممثل خاص ليتولى حشد الاهتمام. هناك ثلاثة عناصر تتعلق بمشكلة الأدوية المضادة للميكروبات يمكن علاجها من خلال فرض السياسات؛ ألا وهي ضمان تيسير سبل الحصول على الدواء، والحفاظ على النتائج، والتفكير الخلاق. ومن أجل التعامل مع كل هذه العناصر الثلاثة، فإنه يلزم التوصل إلى التوليفة السليمة من الخيارات وربطها بمحفل قادر على تفعيل النتائج، كما يلزم دعمها من خلال إطار قانوني عالمي.

## 摘要

全球政府监管和市场调节不力，意味着无法保证持续有效抗菌剂药物的获取。许多人在努力克服这些不利因素，但是他们的机构未充分协调、积极性无法充分调动、没有正确的领导，同时缺乏经费支持。想要促进在全球范围内针对抗菌剂的可获得性和效果采取集体行动，可选方案包括：构建体系机构、建立激励机制和调动行动意愿。单独一项方案不足以应对与抗菌剂耐药性相关的挑战。可行的机构方案包括：对里程碑式的重要成果进行监控和在代理商间成立特别任务小组。全球范围内筹集到的资金，可用于建立激励机制和任命主管行动意愿调动相关事项的特别代表。抗菌剂问题由三个政策要素构成--确保可获得性、保存和创新。为了解决这三个构成要素，不同方案的正确组合需要符合相关管辖地法院要求，而且可能需要国际法律体系的支持。

## Резюме

Глобальное управление и неэффективность рыночного механизма обуславливают невозможность обеспечения доступа к противомикробным препаратам с постоянной эффективностью. Многие люди занимаются решением этих проблем, однако они сталкиваются с недостаточным координированием, руководством и финансированием их учреждений и инициатив. Добиться глобальных коллективных действий по обеспечению доступа к противомикробным препаратам и их эффективности можно в том числе путем организации учреждений, создания стимулов и привлечения заинтересованных лиц. Реализации какого-либо одного из предложенных вариантов недостаточно, чтобы решить все задачи, связанные с резистентностью к противомикробным препаратам. Перспективные варианты организационного действия включают в себя отслеживание основных этапов развития и создание межучрежденческой целевой группы. Для создания стимулов может быть задействован мировой объединенный фонд, а ответственным за привлечение заинтересованных сторон может быть назначен специальный представитель. Стратегия по преодолению проблемы противомикробных препаратов включает три составляющие: обеспечение доступа, рациональное использование и инновации. Чтобы направить силы на все эти три составляющие, необходим правильный набор вариантов в сочетании с площадкой для эффективного обсуждения и, возможно, подкрепление в виде международной правовой основы.

## Introduction

Antimicrobial medicines now save millions of lives each year and many infectious diseases are far less deadly because of them.^1^ However, bacteria, viruses, parasites and fungi develop resistance to existing medicines and few novel antimicrobial products are being produced. Antimicrobial resistance – i.e. resistance of microorganisms to an antimicrobial drug that was originally effective for treating the infection it causes – is both natural and inevitable. However, inappropriate antimicrobial use, falsified or substandard drugs and poor infection control accelerate the pace of evolutionary processes.^1^

Today, diminishing antimicrobial effectiveness represents one of the greatest threats to human health.^2^^–^^4^ Annual deaths from drug-resistant infection are projected to increase from 700 000 to 10 million by 2050, at a cumulative cost of 100 trillion United States dollars (US$).^4^^,^^5^ The world might face a scenario where infection once again takes a heavy toll on a scale and severity not seen in over 80 years. Universal access to antimicrobials, on the other hand, represents one of the greatest opportunities to save millions of lives each year and improve the lives of millions more. For example, 244 000 deaths in neonates could be averted annually with basic injectable antibiotics.^3^

Global action is needed to mitigate the threat of increased antimicrobial resistance. However, policies designed to improve access to antimicrobial medicines, to maintain their effectiveness and to increase the supply of new products have not been implemented.^1^ We argue that this lack of action is due to failures in global governance and global markets, rather than insufficient awareness or political priority. National governments would all benefit from cooperation and coordination on antimicrobial access, conservation and innovation, but none want to incur their part of the associated costs.^6^^,^^7^ Global markets, meanwhile, undersupply antimicrobials for those who cannot afford them, oversupply them in wealthier contexts where individual benefits are not weighed against total costs and underinvest in research and development for new antimicrobials.^8^

We examine ways of achieving global collective action to correct these governance and market failures. Overcoming these failures should make it possible to implement policies designed to improve access to antimicrobials, conserve those that are still effective and drive innovation in preventing and treating infections. We map the existing actors in this policy area, identify guiding institutional design principles and evaluate 10 options for achieving progress. Our goal is to bring the science of global strategy^9^ to bear on the challenge of antimicrobial resistance.

### Governance of antimicrobial use

Many institutions address the threat posed by antimicrobial resistance (Box 1), with numerous global strategies, political resolutions and regulatory standards generated from multilateral activities, industry initiatives and public–private partnerships. However, the mandates and objectives of these institutions are not all aligned. For example, antimicrobial growth-promoters can advance Food and Agriculture Organization (FAO) objectives by improving weight gain in farm animals, but can adversely affect human health, of concern for the World Health Organization (WHO). These institutions work through different policy fora which have different powers to influence state behaviour and are attended by different delegations with different priorities. Ministers of agriculture attend FAO meetings, while ministers of health are at WHO. There is no forum in which they meet to resolve issues of common concern – such as antimicrobial resistance – on the international level. Commitments made by ministers of health to address the issue have resulted in several World Health Assembly resolutions (e.g. WHA51.17, WHA54.11, WHA54.14 and WHA58.27) that have not been implemented. In 2007, WHO reported that:

Box 1 **Examples of key institutions in the global antimicrobial regime**

**United Nations entities**

- World Health Organization (WHO)
- Roll Back Malaria Partnership
- STOP TB Partnership
- Joint United Nations Programme on HIV/AIDS (UNAIDS)
- United Nations Children’s Fund (UNICEF)
- United Nations Office on Drugs and Crime
- United Nations Development Programme (UNDP)
- Food and Agriculture Organization of the United Nations (FAO)
- Joint FAO/WHO Codex Alimentarius Commission
- United Nations General Assembly
- United Nations Security Council

**Other multilateral organizations**

- Global Fund to Fight AIDS, Tuberculosis and Malaria
- International Conference on Harmonization of Technical Requirements for Registration of Pharmaceuticals for Human Use
- World Bank group
- World Organisation for Animal Health (OIE)
- International Cooperation on Harmonization of Technical Requirements for Registration of Veterinary Medicinal Products
- Pharmaceutical Inspection Convention and Pharmaceutical Inspection Co-operation Scheme (PIC/S)
- World Trade Organization (WTO)
- Consultative Group on International Agricultural Research (CGIAR)
- G7 and G20
- Global Health Security Initiative

**Civil society**

- Alliance for the Prudent Use of Antibiotics
- Action on Antibiotic Resistance (ReAct)
- Antibiotic Action Team
- Health Action International (HAI)
- Médecins Sans Frontières (MSF)

**Public–private partnerships**

- Innovative Medicines Initiative
- European Platform for the Responsible Use of Medicines in Animals

**Industry groups and professional associations**

- European Federation of Pharmaceutical Industries and Associations
- International Dairy Federation
- International Federation for Animal Health
- International Federation of Pharmaceutical Manufacturers & Associations
- International Hospital Federation
- International Meat Secretariat
- International Poultry Council
- World Farmers’ Organisation
- World Medical Association
- International Pharmaceutical Federation
- World Health Professions Alliance

“…few countries have a national task force or strategy for containment of resistance, a reference laboratory for surveillance, or enforcement of policies such as limiting the availability of antibiotics to prescription only.”^10^

Nonetheless, some progress towards global collective action on antimicrobials has been made in recent years, in areas such as disease surveillance and food safety. Numerous pathogen- and region-specific surveillance networks are supported by WHO. The International Health Regulations require that all WHO Member States monitor and report disease outbreaks. The World Organization for Animal Health (OIE) sets global standards for antimicrobial surveillance programmes.^11^ FAO, WHO and OIE are currently developing an agreed strategy on antimicrobial resistance.^12^^,^^13^ FAO and WHO already cooperate in the Codex Alimentarius Commission, which develops harmonized international food standards that protect consumer health.^14^

However, it is not clear that the promise of these collaborative efforts will be realized. Debates between human and animal health researchers over drivers of antimicrobial resistance have hindered joint efforts;^15^ the global antimicrobial regime lacks clear leadership and remains fragmented.^16^ Real-world achievements have been elusive. Of the 152 OIE Member States that responded to a 2012 survey, only 27% had systems for monitoring antimicrobial usage in animals, as prescribed by the *Terrestrial Animal Health Code*, with implementation lowest in Africa (5%) and the Americas (4%).^17^ A recent systematic review found that use of human antimicrobial medicines without prescription in countries outside northern Europe and North America ranged from 19% to 100%.^18^ The International Health Regulations have potential to improve this situation, but in 2014, 81 Member States requested a second two-year extension to their original June 2012 deadline for attaining minimal core public health capacities. An additional 48 Member States did not communicate their implementation status or intentions.^19^^–^^21^ The World Health Assembly approved a *Global Action Plan on Antimicrobial Resistance* in May 2015, but its full implementation has yet to be funded and is far from guaranteed.^22^

Four institutional weaknesses contribute to the global lack of action on antimicrobial resistance. The first is a governance problem – an absence of effective coordination across the actors working in different sectors to address this challenge. The second is a compliance problem – a gap between the many actions that have been promised by states and the few that have been delivered. The third is a leadership problem – insufficient political will to stop the inappropriate use of antimicrobials in humans and animals. The fourth is a financing problem – insufficient resources to implement antimicrobial policies.

In the absence of consent-based global action, governments may resort to unilateral measures to coerce collective action, such as direct financing, conditionality, import and export bans or sanctions. These approaches may work but they have several disadvantages (Table 1).

**Table 1:** Unilateral options for promoting state action

Action	How it could work	Disadvantages
Direct financing	Governments could fully or partially finance implementation of specific policies or offer rewards for achieving certain milestones.	• Possibly unaffordable option for any one government. • May deepen paternalistic patron–client relationships and disrupt national priority-setting processes.
Conditionality	Donors could condition development aid and other assistance on recipient states implementing specific policies or achieving certain milestones.	• Risks creating a uniform approach that does not appropriately address each state’s circumstances and needs. • Risks a broader backlash as in the case of structural adjustment programmes and tied development aid.
Import/export bans	States could prohibit the import or export of products associated with antimicrobial resistance, such as medicines and livestock, from or to countries without specific policies such as restrictions on antimicrobial use for promoting animal growth.	• Effect would be limited to countries with trading relations (i.e. only 34 countries can currently export meat to the United States). • Could violate WTO agreements if intended to coerce action in the territory of trading partners rather than protect against a risk to domestic consumers.
Sanctions	Institutions could punish states that lack specific policies or have not achieved certain milestones by withdrawing funding, cutting off relations, restricting financial flows, imposing trade barriers, and public shaming.	• Punitive action could result in significant harm to health, economic and social well-being, especially for the most vulnerable. • Risks undermining multilateralism, principles of sovereign equality and international cooperation on other issues. • Could violate WTO agreements.

WTO: World Trade Organization.

### Strengthening institutions

To correct governance gaps and market failures, the global antimicrobial regime can be changed by adding to or reforming three sets of institutional mechanisms: (i) decision-making mechanisms for setting norms, soliciting advice, making decisions, appealing decisions and resolving disputes; (ii) operational mechanisms for administering activities, for raising, managing and spending funds and for financial auditing; and (iii) accountability mechanisms for making commitments, encouraging compliance, promoting transparency, ensuring oversight and learning from experience.^23^

The optimal package of institutional mechanisms would address current weaknesses by offering effective governance, universal compliance, competent leadership and sufficient financing. Past experience and knowledge of international relations, law and political science offer at least six institutional design principles that can guide us. 

First, global institutions are well positioned to serve some functions and not others because governments commit to and comply with international rules for particular reasons. For example, realist scholars argue international relations primarily reflect states’ own rational self-interests and pursuit of wealth, power and status.^24^ Institutionalists believe states cooperate and coordinate to maximize utility under conditions of interdependence.^25^ Liberal theorists suggest that domestic ideas, interests and institutions affect states’ international relations by shaping state preferences.^26^ Constructivists argue that state behaviour is shaped by ideas, including those derived from international engagement.^27^ While these theories sometimes conflict, together they suggest global institutions should advance states’ rational self-interests, address cooperation and coordination problems, empower domestic actors or change ideas about the world. The impact of any function that global institutions serve also depends critically on states perceiving the function to be a legitimate exercise of delegated authority,^28^ having sufficient capacity to change^29^ and being able to internalize international norms into domestic processes.^30^

Second, global institutions addressing antimicrobial access, conservation and innovation should have clear mandates to ensure they maximize benefits, minimize costs, manage risks and balance trade-offs. International activities are not without costs or risks of harm. There are direct costs like staff salaries, meetings, travel, communications, governance structures and management, and indirect opportunity costs and potential risks of paternalism in placing international norms above local priorities.^31^ Global institutions thus need to be cognizant of these costs and risks, maximize existing institutional architecture and work with others to minimize destructive competition and inefficient duplication.

Third, the forum through which global institutions are created or reformed is important. Different fora have different members, mandates and powers that place structural limits on their activities and competence. The choice of forum for international action also matters because different communities and groups work through different international fora.^32^ For example, since the Framework Convention on Tobacco Control was negotiated through WHO, the influence of health authorities was amplified and the tobacco industry was marginalized. The United Nations (UN) General Assembly, alternatively, has facilitated higher-level whole-of-government engagement with the issues raised by human immunodeficiency virus (HIV), noncommunicable diseases (NCDs) and universal health coverage in a way that seems particularly useful for intersectoral challenges.^33^^,^^34^ However, even the most theoretically well-suited fora may sometimes need to be bypassed if they are too slow, inefficient or otherwise ineffective.^35^^,^^36^

Fourth, global institutions must be specifically tailored for the nature of the problems they are created or reformed to solve. Many global institutions are state-centric which means that they primarily involve national governments and depend on them to regulate nongovernmental actors within their territories. More meaningful involvement of civil society, industry and health-care organizations may strengthen functions that depend on them. Although in this case, reliance on coercive regulation – such as restricting access to antimicrobials – means that states must take centre stage.^37^

Fifth, there seems to be an inverse relationship between the strength of international commitment mechanisms and the activities, norms or standards they involve.^38^ This is because agreements are negotiated as a whole, explaining why states regularly adopt treaties – the strongest international commitment mechanism available – then empty them of ambitious content, which they instead reserve for non-binding commitment mechanisms like political declarations and unilateral statements.^38^ For example, regimes governing trade, human rights, disarmament, prisoners of war and money laundering generally rely on different enforcement mechanisms based on the type of problems addressed and the commitments states are willing to undertake (Box 2).^39^ There is no general hierarchy of impact or influence among global institutions. To strengthen global collective action on antimicrobials, the functions sought, the form that follows and the forum of implementation need to be carefully matched.^40^

Box 2 **Examples of accountability mechanisms in existing international regimes**

International trade provides an example of a problem addressed through a reciprocal exchange of benefits among the World Trade Organization’s (WTO’s) Member States. The political economy of trade policy creates incentives for states to protect domestic firms by erecting barriers to trade. This problem is addressed through trade agreements under which parties have made commitments not to impose particular barriers to trade. In the WTO context, these commitments are enforced through a system of dispute settlement that permits one member to bring a claim against another. This system of enforcement relies on reciprocity in the sense that there is a mutual exchange of concessions between members on a reciprocal basis.

Human rights, in contrast, do not create comparable reciprocal interests between state parties in the observance of treaty commitments. There is no mutual exchange of benefits on a reciprocal basis between parties and no comparable interest in an other’s compliance. As such, accountability mechanisms include reporting, monitoring and individual complaint processes.

Disarmament and fair treatment of prisoners-of-war are both goals in which all states have an interest in ensuring adherence to commitments by a single state acting alone. This collective interest explains the importance of independent inspection and verification in disarmament and humanitarian treaties.

Anti-money laundering efforts by the Financial Action Task Force exemplify a problem addressed through non-binding international recommendations that have considerable coercive power shown by the blacklisting of financial institutions in certain countries. This exclusion has incentivized countries to raise standards to continue transacting with financial institutions abroad.

Sixth, global institutions should be designed for political robustness to withstand inequalities in decision-making and diplomacy.^41^ A realistic view is needed on what different actors can and will do both domestically and internationally, whether by choice or limited by domestic regulations, resources and political constraints. This also means supporting institutions that help enact policy, incentives for those with power to act upon them and interest mobilizers to make the case for their implementation.^42^

### Ten policy options

There are many options for global collective action on antimicrobial medicines, ranging from setting implementation milestones,^1^ to providing new financial models,^43^^,^^44^ to creating new structures,^45^ to adopting legally-binding treaties.^7^^,^^46^^,^^47^We present 10 options for achieving global collective action that illustrate the range of what is possible. Each is assessed according to the global institutional weaknesses addressed and the antimicrobial policy imperatives served (Table 2).

**Table 2:** Ten options for achieving global collective action on antimicrobials

Option	Implementation				Institutional weaknesses addressed					Policy imperatives served		
	Decision-making mechanisms	Operational mechanisms	Accountability mechanisms		Governance	Compliance	Leadership	Financing		Access	Conservation	Innovation
**Institution**												
1. ***Monitored milestones***, including setting goals, timelines, indicators, regular reporting, and UN-, industry- or civil society-led transnational advocacy network monitoring	World Health Assembly or UN General Assembly	UN agencies, civil society networks and/or industry groups	Independent review and evaluation, shadow reports and naming and shaming		–	X	–	–		X	X	–
2. ***Code of practice***, including minimum expectations for responsible use efforts, surveillance and research and development investment among willing actors	Political agreement among willing states, such as G8 countries	Informal governmental networks	Naming and shaming		–	X	–	–		–	X	X
3. ***Inter-Agency Task Force***, coordinating UN and civil society groups	Steering committee of agency representatives	Secretariat of lead UN agency	Annual reports		X	–	X	–		X	X	–
4. ***Intergovernmental panel***, involving scientific working groups and regular reports	Government assembly working groups	Technical support units and academic institutions	Annual reports		–	–	X	–		–	X	–
**Incentive**												
5. ***Funding agreement***, including coordinating joint assistance from development agencies and joint calls for proposals from research funders	Contractual agreement between major donors or research funders	Board of major funders and a secretariat	Annual reports, financial audits and domestic litigation		–	–	–	X		X	X	X
6. ***Global pooled fund***, either to finance antimicrobial policies, reward achieving milestones, procure antimicrobials, or promote research and development	Board of key stakeholders and advisory committees	Secretariat and World Bank as fund trustee. Financing from states, charities and industry	Annual reports, financial audits, independent review and evaluation. Loss of benefits		X	X	–	X		X	X	X
7. ***Conditioning benefits or support***, such as imposing input-, activity-, output- or outcome-based criteria for receiving aid, gaining trade advantages or participating in international initiatives	Governing body of multilateral organization and review panel	Secretariat of multilateral organization	Independent review and evaluation and automatic loss of benefits		–	X	–	–		X	X	–
**Interest mobilizer**												
8. ***Special representatives***, to rally interest groups, coordinate advocacy, attract attention and encourage action	World Health Assembly or UN General Assembly appoints representative	Office of the representative	Political pressure, naming and shaming		–	X	X	–		X	X	–
9. ***High level panel***, involving eminent persons raising political prioritization of antimicrobials	World Health Assembly or UN General Assembly appoints panel	Offices of the panel’s chairs or conveners	Political pressure		–	X	X	–		X	X	–
10. ***Multi-stakeholder partnership***, involving an alliance of many actors, working groups and advocacy	Coordinating committee. Surveillance committee	Offices of partnership members	Annual reports, independent review and evaluation		X	X	–	–		X	X	X

UN: United Nations.

Note: Each option was assessed by two of the authors for whether it would be likely to address the four identified problems in the global antimicrobial regime – governance, compliance, leadership and financing – and contribute to advancing the three antimicrobial policy imperatives – access, conservation and innovation. Assessments were reviewed and commented upon by the remaining authors. Disagreements were resolved through discussion.

Options one to four primarily involve building institutions, ranging in formality. The first is for a global governing body to create milestones and indicators that would then be annually monitored.^1^ Like the Millennium Development Goals, milestones can serve as a commitment device and help promote action if actors know they will be regularly assessed, praised for progress and shamed for any lapses. The second option is a code of practice that outlines minimum expectations for willing signatories. Like the Monterrey Consensus on development assistance targets and the WHO Code of Practice on the International Recruitment of Health Personnel, norms can promote compliance through informal governmental networks and the desire to avoid being seen as “bad”. The third option is a UN inter-agency task force that coordinates the activities of the many UN entities working in this policy area and provides clear direction and leadership for stakeholders. Such task forces exist for NCDs, disaster reduction and violence against women. The fourth option is an intergovernmental panel – like the UN Intergovernmental Panel on Climate Change – that marshals available evidence to inform policies on global antimicrobial resistance.^45^^,^^48^

Options five to seven primarily involve crafting incentives. Option five is a funding agreement between development agencies and institutions that can promote antimicrobial access, conservation and/or innovation. Option six is a global pooled fund that allocates contributions from various donors to finance policies, reward milestones achieved or provide incentives for research and development. Option seven is for multilateral organizations to impose conditions on any support that they provide, such as requiring governments to share surveillance data or ensure that their citizens have access to antimicrobial medicines before receiving additional aid, gaining trade advantages or participating in international initiatives.

Options eight to 10 primarily involve mobilizing interests at a range of scales. Option eight is to appoint a special representative, like the UN Human Rights Council’s special rapporteurs or the UN Secretary-General’s envoys, who would use the prestige of their office to rally interest groups, coordinate advocacy, attract attention and encourage action. Option nine is to appoint a high-level panel of eminent persons that would use their access to people in power to apply political pressure. Option 10 is a multi-stakeholder partnership, like the UN Secretary-General’s Every Woman Every Child movement, which involves an alliance of many actors, working groups and advocacy across fora.

While each option has its merits, none is individually sufficient. Instead, multiple options will need to be adopted – with global decision-makers able to mix-and-match, hopefully in a way that builds on comparative advantages. As a starting point, the optimal package of options probably includes at least one from each of the three categories: institutions, incentives and interest mobilizers.^42^ Within the institutional options, monitored milestones and an inter-agency task force seem most promising, especially given the failure of previous codes of practice,^49^ including those involving antimicrobial medicines.^17^^,^^50^ Existing mechanisms to achieve scientific consensus in medicine and public health probably make a big intergovernmental panel unnecessary.^48^ For incentives, a global pooled fund seems to dominate the other options. Funding agreements address few of the political economy challenges faced and it could be unethical to put conditions on support given to states. Appointing a special representative seems the most practical option for mobilizing interests. A special representative could achieve similar outcomes to a far costlier high-level panel and is more feasible than a multi-stakeholder partnership.

### An international legal framework

In addition to options for building institutions, crafting incentives and mobilizing interests, an international legal framework for antimicrobial resistance could be used to combine different options.^46^ Of the many global health challenges for which treaties have been proposed, the problem of antimicrobial resistance is a strong candidate for an international treaty.^38^^,^^51^

Support for an international legal framework is justified given that antimicrobial resistance is a major transnational risk involving the global exploitation of an essential common resource for which legal instruments have a reasonable chance of achieving benefits and alternative commitment mechanisms have thus far proven ineffective.^38^^,^^51^ Like the legs of a tripod, each antimicrobial policy imperative – access, conservation and innovation – requires a strong, simultaneous level of support from the other two. This is because the policy imperatives are mutually reinforcing: untreated infections spread resistance and the size of market is smaller when many people have no access to antimicrobials; resistance diminishes the value of access to existing antimicrobials and puts a time-limit on their sale; and innovation needs both appropriate access and conservation policies to ensure there can be a return on investment.^46^ An international legal framework may be the best way to achieve progress on all three components at once.^47^

### Fora for implementation

If decision-makers take action, they must decide whether to reform existing global institutions or to create something new. From a policy perspective, it is appealing to create stand-alone initiatives either under sponsorship of an existing organization or through a new forum. WHO is the most obvious existing organization, especially given its unusually expansive powers for making new international treaties under Articles 19 and 21 of its Constitution. Yet WHO’s current financing and governance challenges indicate that an alternative forum may be needed.^36^^,^^52^ Alternatives include bodies like FAO, OIE, the UN General Assembly and UN Security Council, or smaller groupings like the G7/G8, G20, G77, or the Oslo-7 Foreign Policy and Global Health countries.^40^ Other platforms, like the UN Office for Disarmament Affairs and the Biological Weapons Convention, could also be relevant for specific functions such as antimicrobial surveillance as they have become increasingly important fora for addressing infectious disease threats.

From a political economy perspective, stand-alone initiatives may not be possible. Institutions, incentives and interests may not coalesce into a workable package of policy prescriptions and implementation mechanisms. The momentum generated by existing institutions, incentives in other policy areas and interest mobilizers may need to be harnessed. Incorporating policies and mechanisms into existing platforms may help overcome the high threshold for starting something new while simultaneously facilitating cross-forum bargaining. Such incorporation will influence the final policies adopted, depending on how decisions are made, who is involved, which actors dominate, where priorities lie, and pre-existing informal bargains. Rules made through sector-based fora will naturally favour the relevant sector.^40^

## Conclusion

Despite considerable challenges and a history of inaction on antimicrobial resistance, progress should be possible if policy options are matched with the right forum that aligns institutions, incentives and interests towards global collective action. What is needed is a commitment to action and implementation of the many recommendations that have already been made, especially WHO’s *Global Action Plan on Antimicrobial Resistance*.^22^ Global decision-makers must now combine the science of strategy with the art of the possible. Preserving and continuing advances in global health depend on doing so.
